# Clinical Outcome Assessments in Movement Disorders: A Scoping Review of Current Rating Scales and Future Needs Beyond Parkinson's Disease

**DOI:** 10.1002/mdc3.70890

**Published:** 2026-11-06

**Authors:** Panagiota Tsitsi, Evita Papathoma, Nirosen Vijiaratnam, Camila Aquino, Stephen R. Duma, Norbert Kovacs, Kigocha Lameck Okeng'o, Aparna Wagle Shukla, Roongroj Bhidayasiri, Alberto J. Espay, Tiago A. Mestre, Alvaro Sanchez Ferro, Michelle H.S. Tosin, Matej Skorvanek, Ioanna Markaki

**Affiliations:** ^1^ Department of Clinical Neuroscience Karolinska Institutet Stockholm Sweden; ^2^ Department of Neurology Onze‐Lieve‐Vrouw Hospital Aalst Belgium; ^3^ Department of Neurology Karolinska University Hospital Stockholm Sweden; ^4^ Department of Neurology Monash Health Melbourne VIC Australia; ^5^ Clinical Research Centre for Movement Disorders and Gait, Kingston Centre Parkinson's Foundation Centre of Excellence, Monash Health Melbourne VIC Australia; ^6^ Department of Clinical Neurosciences Cumming School of Medicine, Hotchkiss Brain Institute, University of Calgary Calgary AB Canada; ^7^ Department of Neurology St George Hospital Sydney NSW Australia; ^8^ St George and Sutherland Clinical School UNSW Medicine Kogarah NSW Australia; ^9^ Department of Neurology Medical School, University of Pécs Baranya Hungary; ^10^ Muhimbili National Hospital Dar es Salaam Tanzania; ^11^ University of Florida Gainesville FL USA; ^12^ Department of Medicine, Chulalongkorn Centre of Excellence for Parkinson's Disease and Related Disorders Faculty of Medicine, Chulalongkorn University and King Chulalongkorn Memorial Hospital, Thai Red Cross Society Bangkok Thailand; ^13^ The Academy of Science, The Royal Society of Thailand Bangkok Thailand; ^14^ Department of Neurology, James J. and Joan A. Gardner Center for Parkinson's Disease and Movement Disorders University of Cincinnati Cincinnati OH USA; ^15^ Parkinson's Disease and Movement Disorders Clinic, Division of Neurology, Department of Medicine The Ottawa Hospital Research Institute, University of Ottawa Brain and Mind Institute Ottawa ON Canada; ^16^ Movement Disorders Unit, Neurology Department Hospital Universitario 12 de Octubre Madrid Spain; ^17^ Centro de Investigacion Biomédica en Red sobre Enfermedades Neurodegenerativas (CIBERNED) Madrid Spain; ^18^ Department of Neurological Sciences Rush University Medical Center Chicago IL USA; ^19^ Department of Neurology University of Pavol Jozef Safarik in Kosice Košice Slovakia; ^20^ Department of Neurology University Hospital of L. Pasteur Košice Slovakia; ^21^ Center of Neurology, Academic Specialist Center Stockholm Sweden

**Keywords:** clinical outcome assessment, dystonia, movement disorders, psychometrics, tremor

## Abstract

**Background:**

Clinical outcome assessments (COA) are essential for evaluating symptom severity, treatment response, and disease progression in movement disorders. As clinical knowledge evolves, it is necessary to ensure COAs continued relevance and validity.

**Objectives:**

The aims were to provide an updated overview of existing Movement Disorder Society (MDS)–recommended COAs in movement disorders and to identify specific constructs or COAs requiring further revision or validation to inform current clinical practice and research.

**Methods:**

The Scientific Evaluation Committee of the MDS‐COA program commissioned a position paper on the current evidentiary state of COAs. A panel of experts conducted a 2‐step review: first, extracting data from previous MDS publications on COA appraisals using a standardized template; second, conducting a scoping review up to May 2026 to identify new COAs or updated validation data in movement disorders. All study designs were considered.

**Results:**

Of the 13 constructs identified in movement disorders other than PD, updated reviews are most urgently warranted for tremor and dystonia, given the volume of new evidence, emergence of novel instruments, and expanding therapeutic options. For all remaining constructs, important advances were identified alongside persistent gaps in validation, longitudinal data, and clinical interpretability.

**Conclusions:**

The principal challenge in movement‐disorder outcome assessment has shifted from developing new instruments to establishing their comparative validity, responsiveness, and clinical interpretability. Tremor and dystonia have accumulated sufficient new evidence to justify updated MDS reviews, whereas across all constructs the major unmet needs are harmonization of existing measures, longitudinal validation, and integration of patient‐reported and digital outcomes into clinically meaningful assessment frameworks.

Clinical outcome assessments (COA) are essential for evaluating symptom severity, treatment response, and disease progression in movement disorders. The Movement Disorder Society (MDS) has long supported the review, validation, and recommendation of COAs through its COA program, and regular reevaluation is crucial as clinical knowledge and treatments evolve. This review evaluates current COA recommendations across ataxia, Huntington's disease (HD), dystonia, tremor, tics, progressive supranuclear palsy (PSP), restless legs syndrome (RLS), and drug‐induced movement disorders (DIMD). It (1) summarizes existing recommended COAs, (2) reviews new evidence per construct, and (3) highlights areas needing revision, to inform clinical practice, research, and future resource allocation.

## Patients and Methods

The Scientific Evaluation Committee (SEC) of the MDS‐COA program commissioned a position paper on the current state of COA recommendations. The review followed a 2‐step process as follows:

### Step 1: Review of the MDS Database of Publications of COA Appraisal in Movement Disorders Other Than Parkinson's Disease

A focused literature search of the database of publications on COA critiques and recommendations (https://www.movementdisorders.org/MDS/MDS‐Rating‐Scales/Rating‐Scales‐Criti.org‐and‐Recommendations.htm) was conducted in March 2024. Using a standardized template, 2 independent reviewers identified the constructs and extracted relevant data from COAs that were recommended, suggested, or listed based on the established MDS‐COA program criteria.^1^ Discrepancies were discussed at consensus meetings and resolved by agreement between the reviewers.

### Step 2: Scoping Review

A scoping review was conducted for each construct to identify new COAs or updated clinimetric data. Searches were performed in PubMed and Web of Science databases. PubMed was selected for its coverage of the biomedical and clinical literature, and Web of Science for complementary multidisciplinary coverage, including journals outside clinical neurology, in which digital and wearable outcome measures are reported. Relevant review articles identified through the searches were additionally examined for eligible primary studies. The search algorithm used combinations of the following terms: disease or disorder name, scale‐related terms (eg, rating scale, questionnaire, clinical outcome assessment), and psychometric terms (eg, validation, reliability, validity, responsiveness, sensitivity to change) (details in Table SS1). For each construct, the search covered publications from January 1 of the year of publication of the corresponding MDS review through May 3, 2026. Studies involving adults or children, regardless of study design that used COAs to assess symptoms in a specific movement disorder, were included. No language restriction was applied at the search stage. Records published in languages other than English were screened using the English‐language title and abstract provided by the indexing database and were retained where these established eligibility and reported the relevant clinimetric data. Formal translation of full texts was not undertaken. Relevant publications were imported into the Rayyan^2^ application for screening. Each construct was allocated to 1 reviewer. Across all constructs, the same predefined eligibility criteria were applied; records for which eligibility was uncertain were referred to a second reviewer and, where uncertainty persisted, discussed within the writing group; and the final set of included studies for each construct was verified by the coordinating authors and reviewed by the SEC prior to synthesis. The literature search and study selection process results are illustrated in a Preferred Reporting Items for Systematic reviews and Meta‐Analyses (PRISMA) flow diagram.^3^

Consistent with the evidence‐mapping purpose of this scoping review, methodological quality assessment of individual studies was not performed.^4^, ^5^

### Step 3: Summary of Updated COA Overview

The updated overview of existing MDS‐recommended COAs is described by movement disorder and construct in a predefined narrative structure (construct, current state, gaps identified, new evidence, conclusions and future directions). The decision to recommend an updated review for each construct was based not on the volume of new publications alone but on a judgment of whether accumulated evidence—new instruments, validation data, or replication studies—is likely to alter existing MDS recommendation levels, from listed to suggested or suggested to recommended.

COA abbreviations are listed in Table SS2.

## Results

The 13 constructs identified in step 1 yielded 2220 studies in step 2. After duplicates were removed, 1777 studies were screened. A full‐text review excluded 616 studies that did not meet the eligibility criteria, resulting in the inclusion of 261 studies (Fig. SS1). Constructs are presented by disease entity (ataxia, HD, dystonia, PSP, RLS, DIMDs) and, where applicable, by symptom domain (behavior, cognition, motor, quality of life [QoL]).

### Construct: Ataxia

#### Current State

In 2021, an MDS Task Force reviewed 14 COAs for ataxia—9 clinician‐rated scales and 5 functional tests—concluding that no single tool is suitable across all ataxia populations and that instrument selection should be disease specific^6^ (Table 1).

**TABLE 1:** Summary of MDS‐recommended clinical outcome assessments for ataxia and task force recommendations

Construct	Last MDS review year	Recommended COAs	Limitations identified	New developments since last review	Task force recommendations 2026	Need for updated review
Ataxia	2021	Scales				
		SARA	Limited assessment of patients in the more severe end of the spectrum and children	Further linguistic validation; applied across various populations: modified version (fSARA); pediatric adaptation; established MCID	Independent validation of newly developed, composite scales, PROs, remote and digital assessments. Better assessment tools for pediatric and severely affected populations. Integration of nonmotor outcomes into standard batteries. Comprehensive longitudinal validation of PROs. Standardization and reliability testing of remote assessments. Better assessment tools for pediatric and severely affected populations. Formal validation pathways for digital biomarkers.	No
		Cooperative Ataxia Rating Scale (ICARS)	Limited assessment of patients in the more severe end of the spectrum and children	Further linguistic validation; applied across various populations		
		FARS for FA	Limited assessment of patients in the more severe end of the spectrum and children			
		UMSARS for MSA				
		Functional tests				
		Ataxia functional composite score				No
		Composite cerebellar functional severity score				
		SCA functional index for SCAs				

Abbreviations: MDS, Movement Disorder Society; COA, clinical outcome assessment; SARA, Scale for the Assessment and Rating of Ataxia; f‐SARA, modified (functional) Scale for the Assessment and Rating of Ataxia; MCID, minimal clinically important difference; PRO, patient‐reported outcome; ICARS, International Cooperative Ataxia Rating Scale; FARS, Friedreich's Ataxia Rating Scale; FA, Friedreich ataxia; UMSARS, Unified Multiple System Atrophy Rating Scale; MSA, multiple system atrophy; SCA, spinocerebellar ataxia.

#### Gaps Identified

Major limitations include poor applicability to severely affected and pediatric populations, as well as ceiling and floor effects. The Ataxia Global Initiative Working Group highlighted priorities: optimizing the Scale for the Assessment and Rating of Ataxia (SARA), expanding patient‐reported outcomes (PRO), improving sensitivity to change, and defining clinically meaningful change thresholds.^7^

#### New Evidence

SARA remains the most widely used COA, translated into multiple languages^8^, ^9^, ^10^, ^11^ and applied across lysosomal disorders,^12^ autosomal recessive spastic ataxia of Charlevoix‐Saguenay (ARSACS),^13^ stroke,^14^ multiple sclerosis,^11^ ultrarare autosomal recessive cerebellar ataxias,^15^ and pediatric populations, alone^16^ and in combination with the International Cooperative Ataxia Rating Scale (ICARS) domains.^17^ ICARS^18^ and BARS^19^ have similarly undergone linguistic validation.

Large natural history studies have shown variability in SARA responsiveness across disease stages, genotypes, and spinocerebellar ataxia (SCA) subtypes.^20^, ^21^, ^22^, ^23^ Rare disease‐specific scales support niche populations but remain disconnected from broader frameworks.^24^, ^25^ The modified (functional) Scale for the Assessment and Rating of Ataxia (f‐SARA) shows improved content validity and psychometric properties in SCAs,^26^, ^27^ though item‐level analyses indicate unequal contributions to sensitivity and no consensus on an optimized version.^22^

Several composite tools have emerged: Spastic Ataxia Composite (SPAXCOM), combining SARA, SPRS, and Friedreich's Ataxia Rating Scale (FARS)‐ADL (activities of daily living), showed improved 2‐year sensitivity but lacked external validation^28^; SCACOMS, integrating CGI‐I, FARS functional staging, and f‐SARA, demonstrated sensitivity in early SCAs and may outperform single scales,^29^ with patient‐perspective analyses broadly supportive despite item‐weighting limitations.^30^, ^31^ FXTAS‐RS and PRAIS are in early validation with no independent replication.^32^, ^33^

PRO development has accelerated: Patient‐Reported Outcome Measure (PROM)‐Ataxia has undergone longitudinal validation in SCAs, demonstrating sensitivity to progression,^34^ and has been translated into Chinese, Italian, and Brazilian Portuguese,^35^, ^36^, ^37^ though most lack longitudinal or treatment‐sensitivity evaluation. PRAIS and FACR‐HI introduce conceptual fragmentation without consensus on a preferred endpoint.^38^

Functional assessments remain under‐integrated: FARS‐ADL exhibits strong psychometric and content validity in SCA1 and SCA3,^39^, ^40^ whereas DGI and ARAT are applied inconsistently.^41^, ^42^ In Friedreich ataxia, mFARS endpoints have gained traction in clinical trials and regulatory contexts, supported by established test–retest reliability of FARS,^43^ demonstrating construct validity, sensitivity to change, and minimal important change for FARS‐ADL across multiple subtypes,^44^ and discriminative validity of digital gait measures against healthy controls.^45^ Efforts to harmonize SARA, mFARS, and ICARS scores in Friedreich ataxia have yielded interconversion formulas enabling cross‐study and meta‐analytic comparisons, though axial subscores show scale‐dependent sensitivity at different disease stages.^46^ Together, these data represent a strengthened psychometric foundation for FARS‐based instruments, with early harmonization work between FARS‐based and SARA/ICARS scoring offering a model for similar efforts across other ataxia subtypes.

Remote assessment has expanded: SARAhome exhibited strong correlation with in‐person scoring but also intraindividual variability,^47^ confirmed across multiple time points in a multicenter SCA3 study.^48^ Additional platforms enable real‐time video‐based evaluation,^49^, ^50^ including in Friedreich ataxia^51^; early comparability has been shown in other populations such as multiple sclerosis,^52^ and ARSACS,^53^, ^54^ though equivalence to in‐person assessment and generalizability remain uncertain. Emerging instruments address previously underrepresented nonmotor domains, including Scale for Ocular Motor Disorders in Ataxia (SODA),^55^ the Cerebellar Impulsivity‐Compulsivity Scale,^56^ and the modified INAS, applied longitudinally in Friedreich ataxia over 12 years to characterize non‐ataxia features beyond cerebellar symptoms^57^; these tools are not yet integrated into standard assessment batteries, and the Cerebellar Cognitive Affective Syndrome Scale remains excluded as it does not directly measure ataxia symptoms.^58^ Wearable sensors, smartphone‐based tools, and machine‐learning models show promise for gait analysis, kinematic measures, and digital biomarkers across Friedreich ataxia, ARSACS, and SCAs, including a prospective composite digital balance score,^45^, ^59^, ^60^, ^61^, ^62^, ^63^, ^64^, ^65^ and feasible real‐world monitoring via smartphone apps^66^ and wearable tools such as Q‐Motor^67^ though most studies remain exploratory with small samples and limited validation.

A minimal clinically important difference (MCID) for SARA of ~1.5 points has been established using combined anchor‐ and distribution‐based methods,^68^ representing an important advance for trial interpretation.

#### Conclusion and Future Directions

Despite substantial progress, most newly developed composite scales, PROs, and digital tools lack independent replication, longitudinal validation, and standardization, conditions that should be met before a formal updated review can meaningfully revise existing recommendations. The most productive next step is therefore consensus work to harmonize existing instruments and establish validation standards, including better tools for pediatric and severely affected populations. An updated review is not warranted.

### Construct: Behavioral Symptoms in HD

#### Current State

The 2016 MDS review evaluated 27 behavioral COAs across 7 domains in HD, recommending only 3 scales, the Irritability Scale, the Beck Depression Inventory‐II (BDI‐II), and the Hospital Anxiety and Depression Scale (HADS), and exclusively for screening (Table 2). No instrument was recommended for diagnosis, severity grading, longitudinal tracking, or treatment response.^69^

**TABLE 2:** Summary of MDS‐recommended clinical outcome assessments for Huntington's disease and task force recommendations

Construct	Last MDS review	Recommended COAs	Limitations identified	New developments since last review	Task force recommendation 2026	Update needed?
*Behavioral symptoms in HD* (irritability, anxiety, depression, apathy, OCB, psychosis, suicidality)	2016	IS	Screening only; single domain (irritability); not HD specific; no severity, longitudinal, or treatment validation.	SIS (=the IS) factor‐analytic work insufficient for routine use. New multidomain tools address the single‐domain gap: HD‐BQ (strong initial psychometrics; companion report mitigates anosognosia); PBA‐s (Spanish validation; weak early/mild‐stage sensitivity).	External validation; companion or clinician reports for anosognosia; HD‐specific multidomain tools for trials and long‐term monitoring.	No
		BDI‐II	Screening only; not HD specific; depression items overlap somatic/motor features; no longitudinal or treatment validation.	Neuro‐QoL/PROMIS mental‐health PROs validated in HD (reliability, test–retest, 12‐ to 24‐month responsiveness)—key advance since 2016; anosognosia still needs companion or clinician input.		
		HADS	Screening only; not HD specific; no longitudinal or treatment validation.	MDIB captures anxiety‐linked interpretation bias (preliminary). LARS‐s profiles apathy subdomains (partial support; not trial ready).		
Cognition in HD	2017	No COA “recommended”; MoCA “recommended with caveats” (severity), “suggested” (screening).	Insufficient HD‐specific validation; scarce longitudinal and treatment data; limited cross‐cultural evidence; no stage‐sensitive or trial‐ready objective scale; no MCID.	MoCA (Italian) and Chinese UHDRS validated; RBANS domain structure questioned; PD‐CRS shows stage‐differentiated trajectories (upgrade candidate); ECAS promising; SelfCog most mature digital tool; Hi‐DEF/Neuro‐QoL cognition supports PRO endpoints; HD‐CAB gaining trial‐battery support.	Replication; longitudinal validation; MCID estimates; early‐stage sensitivity; validate digital tools; pair a brief objective measure with a functional/PRO outcome.	No
Motor symptoms in HD	2018	Tinetti Mobility Test	Performance‐based mobility/balance; insufficient longitudinal validation; ceiling effects in early/premanifest HD.	HD Digital Motor Score (gait, balance, chorea, tapping)—improved sensitivity, not yet regulatory grade; FALL‐HD validated for fall screening; dual‐task/STEPS ecologically valid but not standard.	Early motor biomarker; validate upper‐limb and digital measures; longitudinal, multidomain integration for early/prodromal trials; prioritize digital motor scoring for appraisal.	No
		Berg Balance Scale	Balance focused; ceiling effects early/premanifest; no clinician‐rated functional/motor scale met severity criteria.	UHDRS‐TMS cross‐culturally validated, inter‐rater agreement improved (dystonia subitems/construct validity unresolved); composite UHDRS and juvenile‐onset modifications proposed.		
		Six‐Minute Walk Test	Walking endurance only; no validated upper‐limb measure; poor integration of motor, functional, and real‐world outcomes.	Upper‐limb tasks (shirt‐buttoning, clinch/upper‐limb token transfer, isometric force) lack longitudinal validation; WHODAS 2.0 validated (disability); speech measures emerging (patient relevant + digital biomarkers).		
Quality of life in HD	2018	SF‐36 (patients)	Generic, not HD specific; anosognosia affects self‐report; uneven stage representation; mostly cross‐sectional.	HD‐specific patient tools: HD‐QoL (across stages), HDQLIFE (late stage/end of life), HD‐PRO‐TRIAD (reliable/valid), HD‐HI (symptom burden, preliminary), SRSQ (social relationships), Neuro‐QoL (reliable, responsive); many overlapping tools; no head‐to‐head comparison.	Longitudinal validation; clinically meaningful change; caregiver integration; cover stigma, family planning, future uncertainty; harmonize overlapping tools.	No
		No tool recommended for caregivers	No recommended caregiver QoL instrument; caregiver outcomes underdeveloped.	HDQoL‐C and short form—strong cross‐cultural validity for caregiver QoL; caregiver outcomes still underrepresented longitudinally.		

Abbreviations: MDS, Movement Disorder Society; COA, clinical outcome assessment; HD, Huntington's disease; OCB, obsessive‐compulsive behaviors; IS, Irritability Scale; HD‐BQ, Huntington's Disease Behavioral Questionnaire; PBA‐s, Problem Behaviours Assessment–short; BDI‐II, Beck Depression Inventory‐II; Neuro‐QoL, Quality of Life in Neurological Disorders; PROMIS, Patient‐Reported Outcomes Measurement Information System; PRO, patient‐reported outcome; HADS, Hospital Anxiety and Depression Scale; MDIB, Movement Disorders Interpretation Bias Scale; LARS‐s, short Lille Apathy Rating Scale; MoCA, Montreal Cognitive Assessment; UHDRS, Unified Huntington's Disease Rating Scale; WHODAS 2.0, World Health Organization Disability Assessment Schedule 2.0; RBANS, Repeatable Battery for the Assessment of Neuropsychological Status; PD‐CRS, Parkinson's Disease–Cognitive Rating Scale; ECAS, Edinburgh Cognitive and Behavioural ALS Screen; SelfCog, digitized cognitive monitoring battery; Hi‐DEF, Huntington's Disease Everyday Functioning scale; HD‐CAB, Huntington's Disease Cognitive Assessment Battery; MCID, minimal clinically important difference; FALL‐HD, Brief Fall Questionnaire for Huntington's Disease; STEPS, Step Test Evaluation of Performance on Stairs; UHDRS‐TMS, Unified Huntington's Disease Rating Scale–Total Motor Score; SF‐36, 36‐Item Short Form Health Survey; HD‐QoL, Huntington's Disease Quality of Life Questionnaire; HDQLIFE, Huntington's Disease Health‐Related Quality of Life; HD‐PRO‐TRIAD, Huntington's Disease Patient‐Reported Outcome–Triad; HD‐HI, Huntington's Disease Health Index; SRSQ, Social Relationships Self‐Report Questionnaire; HDQoL‐C, Huntington's Disease Quality of Life–Carers.

#### Gaps Identified

The primary unmet need is for HD‐specific, multidomain, stage‐sensitive instruments capable of reliably capturing severity and longitudinal change, with adequate attention to disease‐related anosognosia limiting self‐report accuracy.

#### New Evidence

The Movement Disorders Interpretation Bias Scale (MDIB) measures negative interpretation bias linked to anxiety in HD, representing a conceptual advance, though validation remains preliminary.^70^ The short Lille Apathy Rating Scale (LARS‐s) has received partial psychometric support for profiling apathy subdomains but is not yet trial ready.^71^ Quality of Life in Neurological Disorders (Neuro‐QoL) and Patient‐Reported Outcomes Measurement Information System (PROMIS) mental health PROs represent the most significant advance since 2016, demonstrating good reliability, test–retest stability, and responsiveness over 12 to 24 months^72^, ^73^; anosognosia^74^ remains a key limitation requiring companion or clinician input alongside self‐report. The Huntington's Disease Behavioral Questionnaire (HD‐BQ) shows strong initial psychometric properties,^75^ with 2026 companion‐report data indicating utility for mitigating anosognosia‐related underreporting.^76^ The Problem Behaviours Assessment–short (PBA‐s) has been validated in Spanish with good inter‐ and intra‐rater reliability,^77^ though large‐scale clinimetric work suggests limited sensitivity to early or mild behavioral change.^78^ Factor‐analytic work on the SIS in HD remains insufficient for routine use.^79^

#### Conclusions and Future Needs

Behavioral assessment in HD has expanded, but substantial gaps persist: limited independent validation, insufficient early‐stage sensitivity, persistent anosognosia challenges, and incomplete evidence of treatment sensitivity. Future work should prioritize external validation, companion‐ or clinician‐reported input, and multidomain HD‐specific tools suitable for clinical trials. An updated review is not required currently.

### Construct: Cognition in HD

#### Current State

The 2017 MDS review found no cognitive COA meeting criteria for “recommended” status in HD; Montreal Cognitive Assessment (MoCA) was “recommended with caveats” for severity assessment and “suggested” for screening (Table 2).^80^

#### Gaps Identified

Key unmet needs include insufficient HD‐specific clinimetric validation, scarce longitudinal and treatment‐sensitivity data, and limited cross‐cultural evidence. No objective scale demonstrated adequate sensitivity for disease stage or trial readiness.

#### New Evidence

Among established global screeners, MoCA has received Italian validation,^81^ the Chinese Unified Huntington's Disease Rating Scale (UHDRS) demonstrated adequate reliability,^82^ and factor analysis questioned the standard Repeatable Battery for the Assessment of Neuropsychological Status (RBANS) domain structure in HD.^83^ Parkinson's Disease–Cognitive Rating Scale (PD‐CRS) has exhibited validity and reliability for global cognition with established HD‐related mild cognitive impairment and HD dementia cutoffs,^84^ and has demonstrated stage‐differentiated cognitive trajectories in a 2023 comparison study,^85^ positioning it as a leading candidate for future upgrading, though evidence remains largely from 1 group. Edinburgh Cognitive and Behavioural ALS Screen (ECAS) shows promise given its reduced motor demands but requires further HD‐specific replication.^86^

Among novel paradigms, the Maze task demonstrates high 12‐month sensitivity to change, outperforming established progression measures but remaining experimental^87^; LASSI‐L detects early deficits in premanifest HD with limited external replication.^88^ Among digital tools, SelfCog is the most mature, with multilingual validity against classical tests and imaging markers and 1‐year decline detection,^89^ whereas HD‐Mobile^90^ and DAT^91^ remain at early validation stages. HD‐CFRS provides a validated assessment of functional cognitive consequences in symptomatic HD,^92^ and Neuro‐QoL Cognition demonstrates longitudinal sensitivity suitable for PRO trial endpoints.^93^, ^94^ Huntington's Disease Everyday Functioning scale (Hi‐DEF) captures early functional cognitive impact across multiple psychometric studies.^95^, ^96^, ^97^ Huntington's Disease Cognitive Assessment Battery (HD‐CAB) has growing support as a fit‐for‐purpose trial battery.^98^

#### Conclusions and Future Needs

Key gaps include limited independent replication, sparse cross‐cultural and longitudinal validation, absent MCID estimates, and insufficient early‐stage sensitivity. The most defensible interim strategy combines a brief objective cognitive measure with a functional or PRO outcome to capture real‐world relevance and mitigate anosognosia. Although PD‐CRS and select digital tools show promise, evidence remains too preliminary or insufficiently replicated to support a formal updated review at this time.

### Construct: Motor Symptoms in HD

#### Current State

A 2018 MDS review of functional ability measures in HD recommended the Tinetti Mobility Test, Berg Balance Scale, and Six‐Minute Walk Test (Table 2); no functional rating scale met recommendation criteria.^99^

#### Gaps Identified

Key limitations included insufficient longitudinal validation, absence of validated upper‐limb measures, and ceiling effects in early and premanifest HD.

#### New Evidence

Clinician‐rated and composite scales: cross‐cultural validation of the Unified Huntington's Disease Rating Scale–Total Motor Score (UHDRS‐TMS) has confirmed reliability in non‐Western populations,^82^ and inter‐rater reliability analyses show improved overall agreement,^100^ though challenges persist for dystonia subitems and construct validity remains incomplete. Composite and modified scales have been proposed to improve stage sensitivity, including the composite UHDRS^101^ and juvenile‐onset adaptations.^102^

Upper‐limb and functional task assessments: upper‐limb task assessments, including shirt buttoning,^103^ Clinch Token Transfer Test,^104^ Upper Limb Token Transfer Test,^105^ and isometric force assessment,^106^ lack robust longitudinal validation. WHODAS 2.0 (World Health Organization Disability Assessment Schedule 2.0) has been validated in a Polish HD cohort as a general disability measure.^107^ Dual‐task paradigms^108^ and the Step Test Evaluation of Performance on Stairs (STEPS)^109^ offer ecologically valid motor assessment but are not integrated into standard batteries. The Brief Fall Questionnaire for Huntington's Disease (FALL‐HD) has been developed and validated for fall screening in HD.^110^

Digital and technology‐based measures: the HD Digital Motor Score leverages smartphone‐based assessment of gait, balance, chorea, and tapping speed, offering improved sensitivity to change, though validation remains insufficient for regulatory use.^111^ Speech measures are emerging as both patient‐relevant outcomes^112^ and objective digital biomarkers of premanifest HD progression,^113^ though standardization and longitudinal validation remain incomplete.

#### Conclusion and Future Needs

Despite the expansion of tools, key challenges persist: absence of a validated early motor biomarker, insufficient validation of upper‐limb and digital assessments, and poor integration of motor, functional, and real‐world outcomes. Future research should prioritize harmonization, longitudinal validation, and multidomain integration to support clinical trials in early and prodromal HD. An updated review is not yet warranted, though digital motor scoring approaches should be prioritized for systematic appraisal as supporting evidence matures.

### Construct: Quality of Life in HD

#### Current State

The 2018 MDS review found the 36‐Item Short Form Health Survey (SF‐36) as the only recommended patient‐reported QoL instrument; no tool was recommended for caregivers.^114^

#### Gaps Identified

Key concerns included self‐report validity given impaired insight, insufficient clinimetric evidence for several instruments, uneven representation across disease stages, predominantly cross‐sectional data, and underdeveloped caregiver outcomes.

#### New Evidence

HD‐specific QoL instruments have gained further clinimetric evidence: Huntington's Disease Quality of Life Questionnaire (HD‐QoL) has been validated across severity stages across physical‐functional, cognitive, mood, and worry domains^115^; Huntington's Disease Health‐Related Quality of Life (HDQLIFE) provides broader HD‐specific health‐related QoL coverage, including late‐stage and end‐of‐life planning^116^; and Huntington's Disease Patient‐Reported Outcome–Triad (HD‐PRO‐TRIAD) demonstrated strong reliability and construct validity.^117^ However, the field now includes multiple partially overlapping instruments with limited head‐to‐head comparisons, uncertain harmonization, and incomplete responsiveness data. The Huntington's Disease Health Index (HD‐HI) offers patient‐centered symptom burden assessment with preliminary validity data but limited stage‐specific performance evidence.^118^ The Social Relationships Self‐Report Questionnaire (SRSQ) associates perceived social relationships with motor, cognitive, and functional decline, though validation is limited to a single population.^119^ Neuro‐QoL has demonstrated acceptable reliability and responsiveness across physical, mental, and social domains,^120^, ^121^, ^122^, ^123^ though HD‐specific concerns, including genetic burden, stigma, and future uncertainty, remain insufficiently captured. The Huntington's Disease Quality of Life–Carers (HDQoL‐C) and its short form demonstrate strong cross‐cultural validity for caregiver QoL across multiple countries,^124^ though caregiver outcomes remain underrepresented in longitudinal and treatment‐focused research.

#### Conclusions and Future Needs

QoL measurement in HD has progressed substantially, but domains such as stigma, social participation, genetic burden, and future uncertainty remain underrepresented in commonly available instruments. Priorities include longitudinal validation, improved responsiveness data, caregiver outcome integration, and harmonization to support trial readiness. Because of the proliferation of partially overlapping instruments without head‐to‐head comparison, an updated review is not yet warranted until harmonization efforts clarify which HD‐specific measures should be prioritized.

### Construct: Dystonia

#### Current State

In 2013, an MDS Task Force reviewed 36 COAs for dystonia, of which 28 met criteria for full evaluation and 7 achieved “recommended” status, all assessing motor severity (Table 3).^125^

**TABLE 3:** Summary of MDS‐recommended clinical outcome assessments for dystonia and task force recommendations

Construct	Last MDS review year	Recommended COAs (acronym)	Limitations identified	New developments since last review	Task force recommendations 2026	Need for updated review
Dystonia	2013					Yes
Blepharospasm						
		Blepharospasm Disability Index (BSDI)	Focuses primarily on vision‐related disability, does not assess dystonic motor features; limited sensitivity to mild impairment/small clinical changes.		Harmonization across phenotypes; coverage of underrepresented phenotypes (laryngeal, oromandibular, pediatric, X‐linked dystonia‐parkinsonism); pediatric tool development; cross‐cultural validation.	
Cervical dystonia						
		Cervical Dystonia Impact Scale (CDIP‐58)	Further validation of responsiveness and refinement of walking subscale needed.			
		Toronto Western Spasmodic Torticollis Rating Scale (TWSTRS)	Complex to administer; lacks clear midline definition, no separate dystonic tremor score, no guidance for rating sensory tricks.	Revised version, further validation.		
Blepharospasm/cervical dystonia						
		Craniocervical Dystonia Questionnaire (CDQ‐24)	Floor effects in the social/family domain, does not measure motor severity.	Further linguistic validation.		
Laryngeal dystonia						
		Voice Handicap Index (VHI)	Not disease specific, further validation in spasmodic dysphonia			
		Vocal Performance Questionnaire (VPQ)	Not disease specific, further validation in spasmodic dysphonia needed, parallel purpose to VHI raises redundancy questions			
Generalized dystonia						
		Fahn–Marsden Dystonia Rating Scale (FMDRS)	Uses weighting that reduces the contribution of eye, mouth, and neck dystonia; lacks detailed rating in different body areas, only subjective scoring for speech and swallowing.	Further validated in children.		

Abbreviations: MDS, Movement Disorder Society; COA, clinical outcome assessment; CDIP‐58, Cervical Dystonia Impact Profile‐58.

#### Gaps Identified

Anatomical coverage is uneven, with trunk, lower‐limb, arm, and task‐specific dystonias lacking adequate tools. Nonmotor features, including sensory symptoms, pain, sleep, cognition, and psychiatric comorbidities, are insufficiently captured. No instrument supports diagnostic differentiation. Pediatric tools are underdeveloped, and implementation barriers include limited training standards and scarcity of validated translations.

#### New Evidence

For blepharospasm, the blepharospasm severity scale has been developed^126^ and validated in a multicenter cohort^127^; the Craniocervical Dystonia Questionnaire‐24 (CDQ‐24) has been translated into Spanish,^128^ and video‐based rating has been explored.^129^ In a comparative study of hemifacial spasm, Tunc's scale was found to be superior for inter‐rater reliability.^130^ For laryngeal dystonia, strong patient–clinician concordance was demonstrated for the USDRS,^131^ the Communicative Participation Item Bank has been validated,^132^ a new Persian Adductor Laryngeal Dystonia–Vocal Symptoms Scale has been developed,^133^ and the Voice Disability Coping Questionnaire has been psychometrically evaluated.^134^ A screening tool for oromandibular dystonia has been introduced.^135^ For cervical dystonia, the Toronto Western Spasmodic Torticollis Rating Scale (TWSTRS) has been further evaluated, including video‐based and avatar‐based versions^136^, ^137^, ^138^, ^139^, ^140^; CCDRS has been further validated^141^, ^142^; Cervical Dystonia Impact Profile‐58 (CDIP‐58) psychometric support has been expanded^143^; LIVECHART PRO has been published and exhibited good reliability^144^; and the Pain in Dystonia Scale (PIDS) has been developed,^145^ with subsequent Italian multicenter validation.^146^ For task‐specific dystonias, PROs for musicians'^147^ and embouchure^148^ dystonia have been developed. The Dystonia Non‐Motor Symptoms Questionnaire (DNMSQuest) has been developed,^149^ translated into German,^150^ and validated for nonmotor screening in generalized dystonia, including a multicenter craniocervical cohort,^151^, ^152^ supporting its utility across focal and generalized phenotypes, including cervical dystonia. DNMSQuest and CDIP‐58 are currently under separate evaluation as part of the MDS nonmotor COA program; findings from that initiative should inform any future dystonia‐specific review. Balance confidence^153^ and video‐based severity ranking^154^ have also been explored. For pediatric dystonia, DIS and DIS‐II have been validated in inherited/idiopathic dystonia and dyskinetic cerebral palsy^155^, ^156^, ^157^; the Hypertonia Assessment Tool has been introduced and validated^158^, ^159^, ^160^; and the Movement Disorder‐Childhood Rating Scale (MD‐CRS), BAD, and age‐related considerations for the Burke‐Fahn‐Marsden have been evaluated.^160^, ^161^, ^162^ For X‐linked dystonia parkinsonism, dedicated COAs have been cross‐culturally validated,^163^, ^164^, ^165^ a differential diagnosis questionnaire has been developed,^164^ and a culturally adapted MoCA has been published.^166^

#### Conclusions and Future Directions

Given the breadth of new evidence spanning motor severity, nonmotor features, pediatric populations, task‐specific subtypes, and remote assessment methods, an updated review is warranted. Remaining priorities include harmonization, coverage of underrepresented phenotypes, pediatric tool development, and cross‐cultural validation.

### Construct: Tremor

#### Current State

In 2013, an MDS Task Force reviewed 32 COAs for tremor screening and severity evaluation, of which 8 met criteria for “recommended” status (Table 4).^167^

**TABLE 4:** Summary of MDS‐recommended clinical outcome assessments for tremor and task force recommendations

Construct	Last MDS review year	Recommended COAs (acronym)	Limitations identified	New developments since last review	Task force recommendations 2026	Need for updated review
Tremor	2013					
Severity						
		Fahn–Tolosa–Marin Tremor Rating Scale	Ambiguous anchor definitions, requires rater training, ceiling effect for upper‐extremity tremor.	Linguistic validation, version for self‐assessment	Advances in tremor treatments highlight persistent gaps in clinician‐used COAs that cannot be fully addressed by PROs alone.	Yes
		Bain and Findley Clinical Tremor Rating Scale	Severity categories rely on subjective impression, poor reliability for voice and kinetic/intention tremor, inconsistencies among raters on the definition and interpretation of kinetic versus intention tremor.	Cross‐scale comparison		
		Bain and Findley Spirography Scale	Subjective ratings, floor and ceiling effects.			
		WHIGET Tremor Rating Scale, version 2	Limited to upper‐extremity tremor, requires rater training, instructions and testing implements require further standardization.			
		TETRAS	Weaker reliability for voice, face, trunk, and leg tremor; lacks full test–retest reliability and responsiveness data for the ADL subscale	PRO version, cross‐scale comparison		
Disability and ADL scales						
		Bain and Findley Tremor ADL Scale	Focuses primarily on upper‐limb function, modest sensitivity to change	Cross‐scale comparison		
Quality‐of‐life scales						
		QUEST	Poor acceptability with high rates of missing data.	Extensive linguistic validation		
Screening						
		WHIGET Tremor Rating Scale, version 1	Stringent diagnostic criteria, published training exists only for version 2, instructions and testing implements require further standardization.			

Abbreviations: MDS, Movement Disorder Society; COA, clinical outcome assessment; PRO, patient‐reported outcome; WHIGET, Washington Heights‐Inwood Genetic Study of Essential Tremor; TETRAS, The Essential Tremor Rating Assessment Scale; ADL, activities of daily living; QUEST, Quality of Life in Essential Tremor Questionnaire.

#### Gaps Identified

No scale has been comprehensively evaluated for sensitivity to change, limiting confidence in detecting meaningful longitudinal or treatment‐related improvements. Test–retest reliability, minimal detectable change, and MCID remain insufficiently defined for most instruments. Inter‐rater reliability depends on rater training, yet training procedures lack standardization. Additional gaps include floor and ceiling effects, limited assessment of non‐upper‐limb tremor, and scarce linguistic validation. The 2013 review focused predominantly on essential tremor; COA coverage for other tremor subtypes, including dystonic, cerebellar, orthostatic, and Holmes tremor, remains insufficient. Furthermore, the distinction between essential and dystonic tremor cannot be reliably resolved even by supervised machine learning, with classification accuracy as low as 29%,^168^ underscoring fundamental diagnostic challenges that outcome measures must contend with. Finally, tremor severity cannot be adequately or interchangeably captured by any single assessment modality; clinician‐rated scales, PROs, performance‐based measures, and digital tools each reflect distinct aspects of tremor burden and are likely all required for comprehensive assessment.

#### New Evidence

TETRAS (The Essential Tremor Rating Assessment Scale)‐PRO has been developed as the first essential tremor‐specific PRO,^169^ followed by the Patient Attainment Scale‐Essential Tremor (PAS‐ET)^170^ and the Most Bothersome Symptom‐Essential Tremor Questionnaire (MBS‐ET),^171^ alongside new measures of tremor‐related stigma^172^ and enfeeblement.^173^ The Quality of Life in Essential Tremor Questionnaire (QUEST) has been validated in French, German, Hungarian, and Turkish^174^, ^175^, ^176^, ^177^; the Fahn–Tolosa–Marin scale in Spanish,^178^ including for patient self‐assessment^179^; and the Essential Tremor Embarrassment Assessment in French.^174^ Spiral‐drawing assessments have been validated as screening tools^180^, ^181^ and severity measures,^182^ and comparative harmonization studies of key tremor COAs have been undertaken.^183^, ^184^, ^185^ Wearable sensor‐based assessments show promise for objective tremor quantification,^186^, ^187^, ^188^, ^189^ remote smartphone‐ or tablet‐based tools demonstrate feasibility for screening and monitoring,^190^, ^191^, ^192^ and video‐based motion analysis has been evaluated as a quantitative tool during magnetic resonance–guided focused ultrasound surgery (MRgFUS) thalamotomy.^193^

#### Conclusions and Future Recommendations

New COAs and additional psychometric validation align with earlier MDS recommendations, but persistent gaps remain in sensitivity to change, standardized rater training, and clinical interpretability. Expanding therapeutic options, including growing use of MRgFUS, and insufficient COA coverage beyond essential tremor together make tremor the highest priority for an updated MDS review. Such a review should broaden scope to include dystonic, orthostatic, cerebellar, and other tremor disorders, and should address whether clinician‐rated scales, PROs, performance‐based measures, and digital tools are interchangeable or complementary in capturing tremor severity.

### Construct: Tics

#### Current State

A 2017 MDS Task Force reviewed 16 severity scales and 13 screening instruments for tic disorders, recommending 5 severity scales (Yale Global Tic Severity Scale [YGTSS], TSCGI, TDS, STSSS, Premonitory Urge for Tics Scale [PUTS]) and 2 screening tools (Motor Tic, Obsessions and Compulsions, Vocal Tic Evaluation Survey [MOVES], A‐TAC).^194^ The YGTSS has since been adopted as the gold standard in European clinical guidelines.^195^

#### Gaps Identified

No single tool captures the full multidimensional nature of tic disorders, including frequency, intensity, complexity, premonitory urges, and functional impairment. Sensory phenomena, contextual variability, and psychosocial impact are inconsistently represented, and validation across age groups, cultures, and clinical settings remains limited.

#### New Evidence

A large‐scale psychometric evaluation confirmed the YGTSS's robustness, with separate reporting of severity and impairment scores preferred over a global composite.^196^ Revisions have been proposed,^197^ with validity supported in parent‐reported formats^198^ and multiple cultural settings.^199^ PUTS has been translated and validated in Korean, Chinese, and Spanish populations,^200^, ^201^, ^202^ with reliable use in individuals older than 10 years,^203^ though utility in younger children remains limited.^204^ The IPUTS was introduced to capture interindividual variability^197^ and has been validated in Chinese.^205^ MRVS provides a shorter practice‐oriented tool with good correlation to YGTSS.^206^ PTQ has demonstrated treatment‐response utility^207^ and has undergone cross‐cultural validation in Chinese populations.^208^, ^209^ MOVES has been validated in French^210^ and Chinese^211^ contexts. New instruments address previously underrepresented domains: stigma (TDSC),^212^ sensory phenomena (SPAS),^213^ and self‐injurious behaviors (Self‐Injurious Behavior Scale for Tic Disorders [SIBS‐T]).^214^ Machine learning and computer vision approaches show promise for automated tic detection from video,^215^, ^216^, ^217^ with best‐practice methodology for video‐based coding now established.^218^ The CGI‐S has been evaluated for tic severity but has not demonstrated superiority over the YGTSS.^219^

#### Conclusions and Future Directions

The field has progressed through refinement of established scales and expansion into new domains, but fragmentation remains a key limitation. Priorities include harmonized multidimensional frameworks, comprehensive validation across age groups and cultures, integration of clinician‐reported outcomes and PROs, and establishment of longitudinal responsiveness and clinically meaningful change thresholds, particularly for digital tools and newly developed instruments targeting stigma, sensory features, and self‐injurious behavior. Given the lack of harmonization and the early validation stage of most new instruments, an updated review is not yet warranted, though this should be revisited once digital and underrepresented‐domain tools mature.

### Construct: PSP

#### Current State

In 2015, the MDS Task Force reviewed 27 COAs for PSP and recommended 3 instruments—one each for global severity (Progressive Supranuclear Palsy Rating Scale [PSPRS]), motor evaluation (Unified Parkinson's Disease Rating Scale [UPDRS]), and cognition (FAB) (Table 5).^220^

**TABLE 5:** Summary of MDS‐recommended clinical outcome assessments for progressive supranuclear palsy and restless legs syndrome, and task force recommendations

Construct	Last MDS review year	Recommended COAs	Limitations identified	New developments since last review	Task force recommendations 2026	Need for updated review
PSP	2015	PSPRS	Requires examiner training; inter‐rater reliability was evaluated using a limited videotaped version lacking several items; further validity assessment required.	Further validation by 1 group and multiple scale modifications published.	Further refinement of existing PSP scales should prioritize strengthening psychometric properties. In addition, development and validation of a PSP‐specific instrument targeting nonmotor symptoms is recommended, as these domains remain insufficiently represented despite their clinical importance.	No
		UPDRS III	Does not adequately assess PSP‐specific features as oculomotor, bulbar, or cognitive impairment; limited relationship between most factor scores and disease stage; further validation studies needed.			
		Frontal Assessment Battery	Performance may be confounded by motor or communication impairments common in PSP; existing inter‐rater reliability data are limited and based on small samples; requires further PSP‐specific validation.			
RLS						
Diagnosis	2014	Hening Telephone Diagnostic Interview			Validation designs, systematic inclusion of mimic populations, development of consensus‐based diagnostic tools for augmentation, cross‐cultural harmonization, and pediatric‐specific diagnostic instruments.	No
		Cambridge–Hopkins Diagnostic Questionnaire for RLS (CH‐RLSq)	No diagnostic instrument for pediatric RLS	Translations and cross‐cultural adaptations of the (CH‐RLSq)		
		RLS Diagnostic Index (RLS‐DI)				
Severity	2014	International Restless Legs Syndrome Study Group Rating Scale (IRLS)	Limited validation for pediatric RLS severity	IRLS: translations and cross‐cultural validations	Pediatric measures	
		Augmentation Severity Rating Scale			PROM optimization	
		Self‐administered IRLS not yet validated at the time, yet recommended	Need for a validated self‐administered version of the IRLS	Self‐administered IRLS validated as a PROM	Digital monitoring	

Abbreviations: MDS, Movement Disorder Society; COA, clinical outcome assessment; PSP, progressive supranuclear palsy; PSPRS, Progressive Supranuclear Palsy Rating Scale; UPDRS III, Unified Parkinson's Disease Rating Scale, Part III (Motor Examination); RLS, restless legs syndrome; PROM, Patient‐Reported Outcome Measure.

#### Gaps Identified

The PSPRS requires further evaluation of content validity, scaling properties, and responsiveness, whereas the UPDRS and FAB lack adequate PSP‐specific validation. Comprehensive tools that address nonmotor and behavioral features are absent, most instruments are English only, and evidence on clinically meaningful change is limited.

#### New Evidence

Additional PSPRS validation and proposed modifications have been reported.^221^, ^222^ The PSP Quality of Life Questionnaire has undergone further validation, including cross‐sectional and longitudinal evaluation of the short and long versions,^223^, ^224^ and PQoL Carers has been developed^225^ and validated in 2 independent cohorts.^226^, ^227^ Interest in nonmotor features has grown, with clinimetric studies of cognitive and mood instruments in PSP populations,^228^, ^229^, ^230^ and the Cambridge Questionnaire for Apathy and Impulsivity Traits (CamQUAIT) demonstrating good construct validity for apathy, impulsivity, and behavioral assessment across frontotemporal lobar degeneration (FTLD)–related syndromes, including PSP.^231^ The PSP Clinical Deficits Scale has been introduced for clinical deficit monitoring^232^ and shown to accurately reflect functional status and PROs/caregiver‐reported outcomes.^233^ A functional assessment tool for repeat tauopathies^234^ and a PSP‐specific screening questionnaire^235^ have also been published.

#### Conclusions and Future Recommendations

Recent progress is meaningful but substantial gaps persist, particularly in PSPRS content validity and responsiveness, PSP‐specific nonmotor and behavioral instruments, and caregiver‐reported outcomes. Although CamQUAIT shows promise for behavioral assessment across FTLD‐related syndromes, a PSP‐specific nonmotor instrument remains an unmet need. The current evidence does not meet the threshold for an updated review.

### Construct: Diagnosis of RLS

#### Current State

In 2014, an MDS‐commissioned review evaluated diagnostic instruments for RLS.^236^ Three tools were recommended (Table 5).

#### Gaps Identified

Most tools were validated against expert clinical diagnoses, which limits generalizability. Progress since 2014 has focused on refining clinical diagnosis rather than establishing a novel gold standard. A validated pediatric RLS diagnostic instrument is still lacking.

#### New Evidence

A major conceptual advance was the 2014 update to the International Restless Legs Syndrome Study Group diagnostic criteria,^237^ introducing a fifth essential criterion requiring the exclusion of alternative medical or behavioral explanations, adding clinical significance and disease‐course specifiers, and unifying pediatric and adult criteria. Multiple translations and cultural adaptations of established instruments have since been published.^238^, ^239^, ^240^ The Single‐Question Screening Tool has been developed and validated in German^241^ and applied in Norwegian population studies.^240^ The Restless Legs Syndrome Screening Questionnaire (RLSSQ), published in 2009 but not included in the 2014 review, demonstrated high sensitivity (97.9%) and specificity (96.2%) across diverse validation cohorts with strong mimic discrimination.^242^ Several new diagnostic tools have been developed in Indian populations to address sociocultural variation and mimic differentiation, including the Indian RLS Diagnostic Questionnaire,^243^ the Modified‐Restless Legs Syndrome Diagnostic Questionnaire (m‐RLS‐DQ),^244^ the Hindi Restless Legs Syndrome (RLS‐H),^245^ and the ARQIP Questionnaire.^246^ Across these studies, however, sample sizes are small, internal consistency is variable, specificity is limited, and independent replication is lacking.

#### Conclusions and Future Needs

RLS diagnostic assessment has expanded mainly through translations and population‐specific tools. Future priorities include robust independent validation, systematic inclusion of mimics, cross‐cultural harmonization, and the development of pediatric‐specific diagnostic instruments. Given the small samples and lack of independent replication of new diagnostic tools, an updated review is not warranted.

### Construct: Severity of RLS

#### Current State

The 2014 MDS‐commissioned review evaluating severity rating scales for RLS identified 2 instruments that were recommended (Table 5).^247^

#### Gaps Identified

Key gaps include the absence of a validated self‐administered International Restless Legs Syndrome Study Group Rating Scale (IRLS) version, limiting feasibility in clinical and research contexts, and insufficient validation of pediatric severity measures.

#### New Evidence

The IRLS has undergone multiple translations and cross‐cultural validations, reinforcing its robustness and international applicability.^248^

The self‐administered IRLS (sIRLS) has now been formally validated as a PRO, demonstrating excellent internal consistency (Cronbach's α = 0.93), good item homogeneity (0.59), and very high agreement with interviewer‐administered scores (intraclass correlation coefficient = 0.94).^249^ Independent adoption by other research groups addresses a major limitation cited in the 2014 review. RLS symptom diaries have emerged as a complementary approach for capturing daily symptom fluctuations and augmentation, reflecting a broader shift toward longitudinal, ecologically valid tools, though adherence challenges remain.^250^

#### Conclusions and Future Needs

The sIRLS validation represents an important methodological advance, addressing the major limitation of the 2014 review. Remaining priorities include pediatric severity validation, optimized PRO formats, and standardized approaches to symptom fluctuation and augmentation monitoring, including diary‐based and digital tools. As pediatric and digital measurement gaps remain unresolved, an updated review is not warranted.

### Construct: QoL of RLS

#### Current State

The 2014 MDS‐commissioned review recommended only the RLS‐QoL (Abetz) for both cross‐sectional assessment and sensitivity to treatment‐related change.^251^

#### Gaps Identified

Uncertainty in diagnostic ascertainment, with many participants recruited from support groups without expert‐confirmed diagnoses, compromised the evidentiary strength of otherwise psychometrically acceptable tools. No QoL instrument exists for pediatric RLS.

#### New Evidence

The Kohnen RLS‐QoL demonstrated high internal consistency, adequate item homogeneity, good test–retest reliability, and responsiveness to clinical change,^252^ yet validation was conducted by the original developers in clinical trial samples without independent replication. The RLS‐QoL (Abetz) has undergone further cross‐cultural validation, including Turkish adaptation.^253^ Broader instruments addressing psychosocial and self‐management dimensions, including the Pearlin Mastery Scale,^254^ Ethos Brief Index,^255^ and RLS Self‐Care Behaviour Questionnaire,^256^ highlight conceptually relevant domains beyond symptom burden, though none have been integrated into standard RLS outcome frameworks.

#### Conclusions and Future Needs

Advances since 2014 have been limited. Future priorities include independent validation of existing instruments; pediatric QoL tool development; consensus on comprehensive QoL domains, including psychosocial and self‐management aspects; and cross‐cultural harmonization. Given the limited and largely nonindependent new evidence, an updated review is not warranted.

### Construct: DIMDs

#### Current State

In 2023, the MDS‐COA Evaluation Committee systematically reviewed 23 outcome measures for DIMDs; none met criteria for “recommended” status, and 7 symptom‐severity scales—3 of which also served as screening tools—were classified as “recommended with caveats.”^257^

#### Gaps Identified

Most instruments lack adequate evidence for internal consistency, inter‐rater reliability, and responsiveness, limiting confidence in their use for longitudinal tracking and treatment evaluation. No single scale captures the full spectrum of DIMD across both hyperkinetic and hypokinetic phenotypes. Continued reliance on the umbrella term “extrapyramidal symptoms” reflects an outdated conceptual framework that obscures clinically distinct syndromes and complicates cross‐study comparisons.

#### New Evidence

Since 2023, a Slovenian translation and further validation of the Drug‐Induced Extrapyramidal Symptoms Scale have been published.^258^ In parallel, video‐based artificial intelligence approaches have demonstrated high diagnostic accuracy for automated detection of tardive dyskinesia, outperforming trained raters on the Abnormal Involuntary Movement Scale.^259^

#### Conclusions and Future Directions

An updated review is not yet warranted, primarily because the 2023 MDS review is too recent for substantial new evidence to have accumulated. The considerable psychometric limitations of existing scales should be addressed through targeted validation studies. Digital and AI‐based tools represent promising complementary approaches for future assessment and monitoring.

## Limitations

This review has several limitations. It was designed as a broad evidence‐mapping and prioritization exercise rather than an exhaustive systematic review of each individual construct. First, screening in step 2 was performed by 1 reviewer per construct, with referral of uncertain records to a second reviewer. This approach may increase the risk of selection error relative to independent dual screening, and relevant studies may therefore have been missed. Second, the searches were limited to PubMed and Web of Science, and studies indexed exclusively in specialized databases such as Embase or APA PsycINFO may have been missed. This risk was partly mitigated by using the previous MDS systematic reviews as the established evidence base, searching from the date of each corresponding review, and examining relevant review articles for eligible primary studies. Third, records published in languages other than English were assessed based on their English title and abstract only, so extraction of measurement properties for those studies may be incomplete. Fourth, consistent with the evidence‐mapping purpose of a scoping review, methodological quality assessment of individual studies was not performed, and included studies are therefore not weighted by quality in the synthesis.

## Conclusion

This review provides an updated overview of COA development across 13 non‐PD movement disorder constructs, with significant advances in digital and remote assessment, PRO expansion, and disease‐specific instrument development. Although the field has shifted from scale development toward validation and implementation, evidence supporting responsiveness and longitudinal performance remains limited across most constructs, underscoring that the primary need is now harmonization rather than new tool creation. Updated systematic reviews are most urgently warranted for tremor, where expanding therapeutic options such as MRgFUS increase the need for COAs with demonstrated sensitivity to treatment‐related change, a property not yet established for most existing instruments, and for dystonia, where new evidence now spans motor severity, nonmotor features, pediatric populations, and task‐specific subtypes. Future progress will depend on harmonizing existing instruments, establishing clinically meaningful change thresholds, and integrating digital and patient‐centered outcomes into fit‐for‐purpose frameworks for clinical trials and practice.

## Author Roles

(1) Research project: A. Conception, B. Organization, C. Execution; (2) Statistical analysis: A. Design, B. Execution, C. Review and critique; (3) Manuscript preparation: A. Writing of the first draft, B. Review and critique.

P.T.: 1A, 1B, 1C, 3A

E.P.: 1A, 1B, 1C, 3B

N.K.: 1C, 3B

A.W.S.: 1C, 3B

S.R.D.: 1C, 3B

R.B.: 1C, 3B

C.A.: 1C, 3B

N.V.: 1C, 3B

K.L.O.: 1C, 3B

I.M.: 1A, 1B, 1C, 3A

A.J.E.: 1C, 3B

A.S.F.: 1C, 3B

M.H.S.T.: 1A, 1B, 1C, 3B

M.S.: 1A, 1B, 1C, 3B

T.A.M.: 1A, 1B, 1C, 3B

## Disclosures

**Ethical Compliance Statement:** Institutional review board approval was not required for this work, as it is a scoping review of previously published literature and involved no human participants or individual patient data. Informed patient consent was not necessary for this work. We confirm that we have read the Journal's position on issues involved in ethical publication and affirm that this work is consistent with those guidelines.

**Financial Disclosures and Conflicts of Interest:** The Scientific Evaluation Committee of the MDS COA program commissioned this position paper. T.A.M. and A.S.F. report, as support for the present manuscript, a stipend as Director of the MDS COA Program. Conflicts of Interest: The authors declare that there are no conflicts of interest relevant to this work. Financial Disclosures (past 36 months): P.T.: self‐employed neurologist, private practice in Merelbeke, Belgium; support for attending meetings and/or travel: EAN Congress Finland 2025, supported by Merz Pharma GmbH. E.P.: support for attending meetings and/or travel: The Swedish Parkinson Foundation, Parkinsonfonden, travel support to attend MDS Congress 2025 (payment made to her institution). N.V.: None. C.A.: grants or contracts: Parkinson Canada (research funding), Biogen (clinical trial contract); consulting fees: Synucure; payment or honoraria: Movement Disorder Society. S.R.D.: payment or honoraria for lectures, presentations, speakers bureaus, manuscript writing or educational events, and support for attending meetings and/or travel: AbbVie, Ipsen. N.K.: None. K.L.O.: None. A.W.S.: grants or contracts: National Institute of Health; consulting fees: VIMA Therapeutics; leadership or fiduciary role: Tremor Research Group. R.B. has received grants from the following agencies: the Thailand Science Research and Innovation (TSRI, Programme Management Unit for Competitiveness, C01F670185); the National Economic and Social Development Council of Thailand, Thailand Centre of Excellence for Life Sciences (TC [ERP] 31/2568); the National Research Council of Thailand (N42A680591, N35E680087); Chulalongkorn University (CE68_028_3000_004); and the Thailand Research Fund. He is also supported by the Royal Society of Thailand, has provided consultancy services, and has received honoraria and lecture fees from industries such as Britannia, Eisai Pharmaceuticals, Ipsen, Teva‐Lundbeck, and Mitsubishi‐Tanabe. Royalties or licenses: Elsevier Academic Press. Leadership or fiduciary role: Asian Oceanian Association of Neurologists (Secretary and Treasurer); The Neurological Society of Thailand (Chair, International Affairs and Research). R.B. holds patents for various innovations, including a laser‐guided walking stick, a portable tremor device, technology for monitoring during the night (NIGHT‐Recorder), an electronic diary for Parkinson’s disease symptoms, as well as the Parkinson insole and shoe, and antichoking mugs. Furthermore, his copyrighted works comprise the application for screening Parkinson’s disease (Check PD), a Parkinson’s mascot, a daily activity diary for Parkinson’s disease, creative dopamine‐themed lyrics, and educational video content focusing on common nighttime and gastrointestinal symptoms associated with Parkinson’s disease. A.J.E. has received grant support from the NIH and the Michael J. Fox Foundation (payments made to institution), and personal compensation as a consultant/scientific advisory board member for Mitsubishi Tanabe Pharma America (formerly Neuroderm), Amneal, Acorda, Acadia, AbbVie, Bial, Supernus (formerly USWorldMeds), NeuroDiagnostics, Inc. (SYNAPS Dx), Intrance Medical Systems, Inc., Merz, Praxis Precision Medicines, Citrus Health, and Herantis Pharma; A.J.E. is Data Safety Monitoring Board (chair) of AskBio and has received publishing royalties from Lippincott Williams & Wilkins, Cambridge University Press, and Springer. He is co‐inventor of the patent “Compositions and methods for treatment and/or prophylaxis of proteinopathies” with which he cofounded REGAIN Therapeutics to fund preclinical studies. He has no financial relationship with the company and has relinquished the right to any personal income from future treatments. A.J.E. owns no stock in any pharmaceutical company with which he has an advisory relationship. T.A.M. reports stipend: Director MDS COA Program; consulting and Advisory Board Membership with honoraria: AbbVie, International Parkinson and Movement Disorder Society, CHDI Foundation/Management, Roche, WAVE, and PTC Therapeutics; grants and research: EU Joint Program‐Neurodegenerative Disease Research, uOBMRI, Roche, Ontario Research Fund, CIHR, the Michael J. Fox Foundation for Parkinson’s Research, Parkinson Canada, Parkinson Research Consortium, and Brain Canada; salary: UOMA. A.S.F. reports receiving a salary from Hospital 12 de Octubre and a stipend as Director MDS COA Program. He holds patents, receives salary, and holds shares in Leuko Labs, Inc./Leuko Imaging Systems. Patents issued: US Patents 12,383,155; 12,216,112; 12,131,217; 11,963,750; 11,860,154; 11,445,980; 10,959,676; 10,506,979. He receives royalties from patent US20150272504A1, Elsevier, and Digital Technologies in Movement Disorders (Book). He has received speaker honoraria from AbbVie, Abbott, Alter, Bayer, Bial, Boston Scientific, Esteve, the European Academy of Neurology (EAN), Italfarmaco, Medtronic, the Movement Disorder Society (MDS), Novartis, Organon, PD Monitor, Roche, the Spanish Society of Neurology (SEN), Stada, Teva, and Zambon. He has served as a consultant or advisor for AbbVie, Boston Scientific, Esteve, Lundbeck, Orion Pharma, Roche, Prim, and Zambon. Participation on an Advisory Board: Hoffman La Roche. He has received research funding from ERA‐NET Horizon 2020 JPCOFUND2 (HESOCARE‐329‐073), the Spanish Research Agency/Instituto de Salud Carlos III (PI22/01177), EIT Health (PDNetGo), NIH (SBIR grant), and the MDS (eDiary); clinical trial agreements: Hoffman La Roche, Zambon, Ferrer, TEVA Pharmaceuticals. Support for attending meetings and/or travel: MDS, IAPDR, SEN LIMPE, Parkinson Study Group. Leadership or fiduciary role: Chair, Movement Disorders Group, Spanish Neurological Society; Director, COA Program at MDS. M.H.S.T. receives a salary from Rush University and is part of a working group supported by the International Parkinson and Movement Disorder Society. Grants or contracts (payments made to Rush University): International Parkinson & Movement Disorder Society; NINDS (1U01NS143225, 1R01NS135614‐01A1, 5R01NS125294); HHS/NIH (PAF08839); CHDI Foundation; NIA (1R01AG059417‐01A1); NeuraLight Inc. Consulting fees: International Parkinson & Movement Disorder Society (payments made to her). Leadership or fiduciary role: International Parkinson & Movement Disorder Society (unpaid). M.S. receives funding from the EU Renewal and Resilience Plan “Large projects for excellent researchers” under grant number 09I03‐03‐V03‐00007, from the Slovak Research and Development Agency under contract number APVV‐22‐0279, and from Vedecka grantova agentura. Over the past 12 months, M.S. has received speakers’ honoraria from AbbVie, Biogen, Desitin, Medtronic, Krka, Medis, IPMDS, Stada, and Sandoz. He is an advisor to AbbVie and Biogen. Consulting fees: AbbVie, Medis, Biogen, Medison, Medtronic, UCB, Stada. Participation on a Data Safety Monitoring Board or Advisory Board: AbbVie, Medis, Biogen, UCB, Stada, Medtronic. Leadership or fiduciary role: Medical Faculty UPJS Kosice; Slovak Neurological Society. I.M. receives research funding from Region Stockholm, Karolinska Institutet and Parkinsonfonden. Payment or honoraria: AbbVie educational seminar. Support for attending meetings and/or travel: AbbVie advisory board (travel and hotel stay). Participation on an Advisory Board: AbbVie (institutional fees). Author disclosures are available in the Supporting Information.

## Supporting information


**Figure S1.** Flowchart diagram for studies included in the review.

**Table S1.** Search algorithms.

**Table S2.** Abbreviations.

**Supplementary S1.** Disclosure statement form.

## Data Availability

Data sharing not applicable to this article as no datasets were generated or analysed during the current study.
