# Supplementary material for: Clinical Outcome Assessments in Movement Disorders: A Scoping Review of Current Rating Scales and Future Needs Beyond Parkinson's Disease
Source: Mov Disord Clin Pract. 2026 Nov 6:10.1002/mdc3.70890. Online ahead of print. doi: 10.1002/mdc3.70890 (PMC13743828; doi:10.1002/mdc3.70890)
Supplement: Supplementary file 1 [file MDC3-9999-0-s001.docx]

**Supplemental Table 1.** Search Algorithms

| **Construct** | **Pubmed** | **Web of Science** |
| --- | --- | --- |
| Ataxia, 2020 | (("Ataxia"[MeSH Terms] OR ataxia[Title/Abstract] OR "cerebellar disorders"[MeSH Terms] OR cerebellar[Title/Abstract]) AND  ("rating scale"[Title/Abstract] OR "clinical scale"[Title/Abstract] OR questionnaire[Title/Abstract] OR assessment[Title/Abstract] OR instrument[Title/Abstract] OR "functional test"[Title/Abstract] OR "Clinical Outcome Assessment"[Title/Abstract] OR COA[Title/Abstract]) AND  (validation[Title/Abstract] OR psychometric[Title/Abstract] OR reliability[Title/Abstract] OR validity[Title/Abstract] OR responsiveness[Title/Abstract] OR "sensitivity to change"[Title/Abstract])) | (ataxia OR "cerebellar disorder*" OR "cerebellar ataxia*" OR cerebellar) AND ("rating scale*" OR "clinical scale*" OR "clinical rating*" OR questionnaire* OR instrument* OR "functional test*" OR "outcome measure*" OR "Clinical Outcome Assessment" OR COA) AND (validation OR psychometric* OR reliability OR validity OR responsiveness OR "sensitivity to change" OR "clinimetric*") (Topic) |
| Quality of life in Huntigton’s Disease, 2018 | ("Huntington Disease"[MeSH Terms] OR "Huntington*"[Title/Abstract]) AND ("quality of life"[Title/Abstract] OR QoL[Title/Abstract] OR "health-related quality of life"[Title/Abstract] OR HRQoL[Title/Abstract]) AND ("scale"[Title/Abstract] OR "questionnaire"[Title/Abstract] OR "assessment"[Title/Abstract] OR "instrument"[Title/Abstract] OR "outcome measure"[Title/Abstract] OR "Clinical Outcome Assessment"[Title/Abstract] OR COA[Title/Abstract]) AND (validation[Title/Abstract] OR psychometric[Title/Abstract] OR reliability[Title/Abstract] OR validity[Title/Abstract] OR clinimetric[Title/Abstract] OR responsiveness[Title/Abstract] OR "sensitivity to change"[Title/Abstract] OR measure[Title/Abstract] OR development[Title/Abstract]) | ("Huntington Disease" OR "Huntington*") AND ("quality of life" OR QoL OR "health-related quality of life" OR HRQoL) AND ("scale*" OR questionnaire* OR assessment* OR instrument* OR "outcome measure*" OR "Clinical Outcome Assessment" OR COA) AND (validation OR psychometric* OR reliability OR validity OR clinimetric* OR responsiveness OR "sensitivity to change" OR development OR measure*) (Topic) |
| Motor symptoms in Huntington's disease, 2018 | ("Huntington Disease" [MeSH Terms] OR "Huntington*"[Title/Abstract]) AND ("motor"[Title/Abstract] OR "movement"[Title/Abstract] OR "motor symptom*"[Title/Abstract] OR "neurological sign*"[Title/Abstract] OR "motor function"[Title/Abstract]) AND ("scale"[Title/Abstract] OR "rating scale"[Title/Abstract] OR "clinical scale"[Title/Abstract] OR "clinical rating"[Title/Abstract] OR "questionnaire"[Title/Abstract] OR "assessment"[Title/Abstract] OR "instrument"[Title/Abstract] OR "outcome measure"[Title/Abstract] OR "Clinical Outcome Assessment"[Title/Abstract] OR COA[Title/Abstract]) AND (validation[Title/Abstract] OR psychometric[Title/Abstract] OR reliability[Title/Abstract] OR validity[Title/Abstract] OR clinimetric[Title/Abstract] OR responsiveness[Title/Abstract] OR "sensitivity to change"[Title/Abstract] OR measure[Title/Abstract] OR development[Title/Abstract]) | (("Huntington Disease" OR "Huntington*") AND (motor OR "motor function" OR "motor symptom*" OR "movement disorder*" OR "neurological sign*") AND ("rating scale*" OR "clinical rating*" OR "clinical scale*" OR questionnaire* OR assessment* OR instrument* OR "outcome measure*" OR "Clinical Outcome Assessment" OR COA OR test*) AND (validation OR psychometric* OR reliability OR validity OR clinimetric* OR responsiveness OR "sensitivity to change")) (Topic) |
| Cognition in Huntington's disease, 2018 | ("Huntington Disease"[MeSH Terms] OR "Huntington*"[Title/Abstract]) AND ("cognition"[Title/Abstract] OR "cognitive"[Title/Abstract] OR "executive function"[Title/Abstract] OR "memory"[Title/Abstract] OR "attention"[Title/Abstract] OR "neuropsychological test*"[Title/Abstract] OR "cognitive symptom*"[Title/Abstract]) AND ("scale"[Title/Abstract] OR "rating scale"[Title/Abstract] OR "clinical scale"[Title/Abstract] OR "clinical rating"[Title/Abstract] OR "questionnaire"[Title/Abstract] OR "assessment"[Title/Abstract] OR "instrument"[Title/Abstract] OR "outcome measure"[Title/Abstract] OR "Clinical Outcome Assessment"[Title/Abstract] OR COA[Title/Abstract] OR "test"[Title/Abstract]) AND (validation[Title/Abstract] OR psychometric[Title/Abstract] OR reliability[Title/Abstract] OR validity[Title/Abstract] OR clinimetric[Title/Abstract] OR responsiveness[Title/Abstract] OR "sensitivity to change"[Title/Abstract] OR measure[Title/Abstract] OR development[Title/Abstract]) | (("Huntington disease" OR "Huntington*") AND ("cognition" OR "cognitive" OR "executive function" OR "memory" OR "attention" OR "neuropsychological test*" OR "cognitive symptom*") AND ("rating scale*" OR "clinical scale*" OR "clinical rating*" OR questionnaire* OR assessment* OR instrument* OR "outcome measure*" OR "Clinical Outcome Assessment" OR COA OR test*) AND (validation OR psychometric* OR reliability OR validity OR responsiveness OR "sensitivity to change" OR clinimetric*)) (Topic) |
| Behavioral symptoms in Huntington's disease, 2016 | ("Huntington Disease"[MeSH Terms] OR "Huntington*"[Title/Abstract]) AND ("behavioral symptom*"[Title/Abstract] OR "behavioural symptom*"[Title/Abstract] OR "psychiatric symptom*"[Title/Abstract] OR "neuropsychiatric symptom*"[Title/Abstract] OR "mood"[Title/Abstract] OR "depression"[Title/Abstract] OR "apathy"[Title/Abstract] OR "irritability"[Title/Abstract] OR "aggression"[Title/Abstract] OR "anxiety"[Title/Abstract] OR "obsessive-compulsive"[Title/Abstract] OR "psychosis"[Title/Abstract]) AND ("rating scale"[Title/Abstract] OR "clinical scale"[Title/Abstract] OR "clinical rating"[Title/Abstract] OR "questionnaire"[Title/Abstract] OR "assessment"[Title/Abstract] OR "instrument"[Title/Abstract] OR "outcome measure"[Title/Abstract] OR "Clinical Outcome Assessment"[Title/Abstract] OR COA[Title/Abstract] OR "test"[Title/Abstract]) AND (validation[Title/Abstract] OR psychometric[Title/Abstract] OR reliability[Title/Abstract] OR validity[Title/Abstract] OR clinimetric[Title/Abstract] OR responsiveness[Title/Abstract] OR "sensitivity to change"[Title/Abstract]) | (("Huntington disease" OR "Huntington*") AND ("behavioral symptom*" OR "behavioural symptom*" OR "psychiatric symptom*" OR "neuropsychiatric symptom*" OR mood OR depression OR apathy OR irritability OR aggression OR anxiety OR "obsessive-compulsive" OR psychosis) AND ("rating scale*" OR "clinical scale*" OR "clinical rating*" OR questionnaire* OR assessment* OR instrument* OR "outcome measure*" OR "Clinical Outcome Assessment" OR COA OR test*) AND (validation OR psychometric* OR reliability OR validity OR clinimetric* OR responsiveness OR "sensitivity to change")) (Topic) |
| Scales for Antipsychotic-Associated Movement Disorders, 2023 | (("Antipsychotic Agents"[MeSH Terms] OR antipsychotic*[Title/Abstract] OR neuroleptic*[Title/Abstract]) AND ("movement disorder*"[Title/Abstract] OR "drug-induced movement*"[Title/Abstract] OR "extrapyramidal symptom*"[Title/Abstract] OR EPS[Title/Abstract] OR "tardive dyskinesia"[Title/Abstract] OR "akathisia"[Title/Abstract] OR "drug-induced parkinson*"[Title/Abstract] OR "parkinsonism"[Title/Abstract] OR "dystonia"[Title/Abstract])) AND ("rating scale"[Title/Abstract] OR "clinical scale"[Title/Abstract] OR "clinical rating"[Title/Abstract] OR "questionnaire"[Title/Abstract] OR "assessment"[Title/Abstract] OR "instrument"[Title/Abstract] OR "outcome measure"[Title/Abstract] OR "Clinical Outcome Assessment"[Title/Abstract] OR COA[Title/Abstract] OR "test"[Title/Abstract]) AND (validation[Title/Abstract] OR psychometric[Title/Abstract] OR reliability[Title/Abstract] OR validity[Title/Abstract] OR clinimetric[Title/Abstract] OR responsiveness[Title/Abstract] OR "sensitivity to change"[Title/Abstract])) | (("antipsychotic*" OR neuroleptic*) AND ("movement disorder*" OR "drug-induced movement*" OR "extrapyramidal symptom*" OR EPS OR "tardive dyskinesia" OR akathisia OR "drug-induced parkinson*" OR parkinsonism OR dystonia) AND ("rating scale*" OR "clinical scale*" OR "clinical rating*" OR questionnaire* OR assessment* OR instrument* OR "outcome measure*" OR "Clinical Outcome Assessment" OR COA OR test*) AND (validation OR psychometric* OR reliability OR validity OR clinimetric* OR responsiveness OR "sensitivity to change")) (Topic) |
| Tics, 2017 | (("Tic Disorders"[MeSH Terms] OR "Tourette Syndrome"[MeSH Terms] OR tic*[Title/Abstract] OR "Tourette*"[Title/Abstract]) AND ("severity"[Title/Abstract] OR "screening"[Title/Abstract] OR "screening instrument*"[Title/Abstract]) AND ("rating scale"[Title/Abstract] OR "clinical scale"[Title/Abstract] OR "clinical rating"[Title/Abstract] OR "questionnaire"[Title/Abstract] OR "assessment"[Title/Abstract] OR "instrument"[Title/Abstract] OR "outcome measure"[Title/Abstract] OR "Clinical Outcome Assessment"[Title/Abstract] OR COA[Title/Abstract] OR "test"[Title/Abstract]) AND (validation[Title/Abstract] OR psychometric[Title/Abstract] OR reliability[Title/Abstract] OR validity[Title/Abstract] OR clinimetric[Title/Abstract] OR responsiveness[Title/Abstract] OR "sensitivity to change"[Title/Abstract])) | TS=(("Tourette syndrome" OR "tic disorder*" OR "motor tic*" OR "vocal tic*")  AND ("severity scale*" OR "rating scale*" OR "clinical rating*" OR "questionnaire*" OR "screening instrument*" OR "assessment tool*" OR "outcome measure*" OR "Clinical Outcome Assessment" OR COA) AND (validation OR psychometric* OR reliability OR validity OR clinimetric* OR responsiveness OR "sensitivity to change")) |
| Clinical features of progressive supranuclear palsy, 2015 | ("Progressive Supranuclear Palsy"[MeSH Terms] OR "Progressive Supranuclear Palsy"[Title/Abstract] OR PSP[Title/Abstract]) AND ("rating scale"[Title/Abstract] OR "clinical rating"[Title/Abstract] OR "clinical scale"[Title/Abstract] OR "questionnaire"[Title/Abstract] OR "assessment tool"[Title/Abstract] OR "instrument"[Title/Abstract] OR "outcome measure"[Title/Abstract] OR "Clinical Outcome Assessment"[Title/Abstract] OR COA[Title/Abstract]) AND (validation[Title/Abstract] OR psychometric[Title/Abstract] OR reliability[Title/Abstract] OR validity[Title/Abstract] OR clinimetric[Title/Abstract] OR responsiveness[Title/Abstract] OR "sensitivity to change"[Title/Abstract] OR review[Title/Abstract] OR "systematic review"[Publication Type] OR "review"[Publication Type]) NOT ("Parkinson Disease"[MeSH Terms] OR Parkinson*[Title/Abstract]) | (("Progressive Supranuclear Palsy" OR PSP) AND ("rating scale*" OR "clinical rating*" OR "clinical scale*" OR questionnaire* OR "assessment tool*" OR instrument* OR "outcome measure*" OR "Clinical Outcome Assessment" OR COA) AND (validation OR psychometric* OR reliability OR validity OR clinimetric* OR responsiveness OR "sensitivity to change")) (Topic) not Parkinson (Topic) |
| Severity of restless legs syndrome, 2014 | ("Restless Legs Syndrome"[MeSH Terms] OR "Restless Legs Syndrome"[Title/Abstract] OR "Willis-Ekbom disease"[Title/Abstract] OR RLS[Title/Abstract]) AND ("severity scale"[Title/Abstract] OR "rating scale"[Title/Abstract] OR "clinical rating"[Title/Abstract] OR "questionnaire"[Title/Abstract] OR "assessment tool"[Title/Abstract] OR "outcome measure"[Title/Abstract] OR "Clinical Outcome Assessment"[Title/Abstract] OR COA[Title/Abstract]) AND (validation[Title/Abstract] OR psychometric[Title/Abstract] OR reliability[Title/Abstract] OR validity[Title/Abstract] OR clinimetric[Title/Abstract] OR responsiveness[Title/Abstract] OR "sensitivity to change"[Title/Abstract]) | ("Restless Legs Syndrome" OR "Willis-Ekbom disease") (Title) and ("severity scale*" OR "rating scale*" OR "clinical rating*" OR "questionnaire*" OR "assessment tool*" OR "outcome measure*" OR "Clinical Outcome Assessment" OR COA) (Title) and (validation OR psychometric* OR reliability OR validity OR clinimetric* OR responsiveness OR "sensitivity to change") (Topic) |
| Diagnosis of restless legs syndrome/Willis-Ekbom disease (RLS/WED), 2014 | ("Restless Legs Syndrome"[MeSH Terms] OR "Restless Legs Syndrome"[Title/Abstract] OR "Willis-Ekbom disease"[Title/Abstract] OR RLS[Title/Abstract] OR WED[Title/Abstract]) AND ("diagnostic instrument*"[Title/Abstract] OR "diagnostic tool*"[Title/Abstract] OR "diagnostic scale*"[Title/Abstract] OR "diagnostic questionnaire*"[Title/Abstract] OR "diagnostic assessment*"[Title/Abstract] OR "screening instrument*"[Title/Abstract] OR "screening tool*"[Title/Abstract]) AND (validation[Title/Abstract] OR psychometric[Title/Abstract] OR reliability[Title/Abstract] OR validity[Title/Abstract] OR clinimetric[Title/Abstract] OR responsiveness[Title/Abstract] OR "sensitivity to change"[Title/Abstract]) | (("Restless Legs Syndrome" OR "Willis-Ekbom disease" OR RLS OR WED) AND ("diagnostic instrument*" OR "diagnostic tool*" OR "diagnostic scale*" OR "diagnostic questionnaire*" OR "diagnostic assessment*" OR "screening instrument*" OR "screening tool*") AND (validation OR psychometric* OR reliability OR validity OR clinimetric* OR responsiveness OR "sensitivity to change" OR review OR "systematic review")) (Title) |
| Quality of life instruments of restless legs syndrome/Willis-Ekbom disease (RLS/WED), 2014 | ("Restless Legs Syndrome"[MeSH Terms] OR "Restless Legs Syndrome"[Title/Abstract] OR "Willis-Ekbom disease"[Title/Abstract] OR RLS[Title/Abstract] OR WED[Title/Abstract]) AND ("quality of life"[Title/Abstract] OR QoL[Title/Abstract] OR "patient-reported outcome*"[Title/Abstract] OR PRO[Title/Abstract] OR "health-related quality of life"[Title/Abstract] OR HRQoL[Title/Abstract] OR "life quality"[Title/Abstract]) AND ("questionnaire"[Title/Abstract] OR "assessment tool"[Title/Abstract] OR "instrument"[Title/Abstract] OR "rating scale"[Title/Abstract] OR "outcome measure"[Title/Abstract] OR "Clinical Outcome Assessment"[Title/Abstract] OR COA[Title/Abstract]) AND (validation[Title/Abstract] OR psychometric[Title/Abstract] OR reliability[Title/Abstract] OR validity[Title/Abstract] OR clinimetric[Title/Abstract] OR responsiveness[Title/Abstract] OR "sensitivity to change"[Title/Abstract]]) | (("Restless Legs Syndrome" OR "Willis-Ekbom disease" OR RLS OR WED) AND ("quality of life" OR QoL OR "patient-reported outcome*" OR PRO OR "health-related quality of life" OR HRQoL OR "life quality") AND ("questionnaire*" OR "assessment tool*" OR instrument* OR "rating scale*" OR "outcome measure*" OR "Clinical Outcome Assessment" OR COA) AND (validation OR psychometric* OR reliability OR validity OR clinimetric* OR responsiveness OR "sensitivity to change")) (Title) |
| Tremor, 2013 | ("Tremor"[MeSH Terms] OR tremor*[Title/Abstract] OR "essential tremor"[Title/Abstract]) AND ("screening instrument*"[Title/Abstract] OR "screening tool*"[Title/Abstract] OR "screening scale*"[Title/Abstract] OR "evaluation scale*"[Title/Abstract] OR "rating scale*"[Title/Abstract] OR "clinical rating"[Title/Abstract] OR "questionnaire"[Title/Abstract] OR "assessment tool"[Title/Abstract] OR "instrument"[Title/Abstract] OR "outcome measure"[Title/Abstract] OR "Clinical Outcome Assessment"[Title/Abstract] OR COA[Title/Abstract]) AND (validation[Title/Abstract] OR psychometric[Title/Abstract] OR reliability[Title/Abstract] OR validity[Title/Abstract] OR clinimetric[Title/Abstract] OR responsiveness[Title/Abstract] OR "sensitivity to change"[Title/Abstract]) NOT ("Parkinson Disease"[MeSH Terms] OR Parkinson*[Title/Abstract]) | ("tremor*" OR "essential tremor") (Title) and ("screening instrument*" OR "screening tool*" OR "screening scale*" OR "evaluation scale*" OR "rating scale*" OR "clinical rating*" OR "questionnaire*" OR "assessment tool*" OR "outcome measure*" OR "Clinical Outcome Assessment" OR COA) (Title) and (validation OR psychometric* OR reliability OR validity OR clinimetric* OR responsiveness OR "sensitivity to change") (Topic) not Parkinson* (Topic) |
| Dystonia, 2013 | ("Dystonia"[MeSH Terms] OR dystonia[Title/Abstract] OR "focal dystonia"[Title/Abstract] OR "cervical dystonia"[Title/Abstract] OR "generalized dystonia"[Title/Abstract] OR "blepharospasm"[Title/Abstract] OR "writer's cramp"[Title/Abstract]) AND ("rating scale"[Title/Abstract] OR "clinical rating"[Title/Abstract] OR "clinical scale"[Title/Abstract] OR "questionnaire"[Title/Abstract] OR "assessment tool"[Title/Abstract] OR "instrument"[Title/Abstract] OR "outcome measure"[Title/Abstract] OR "Clinical Outcome Assessment"[Title/Abstract] OR COA[Title/Abstract]) AND (validation[Title/Abstract] OR psychometric[Title/Abstract] OR reliability[Title/Abstract] OR validity[Title/Abstract] OR clinimetric[Title/Abstract] OR responsiveness[Title/Abstract] OR "sensitivity to change"[Title/Abstract]) NOT ("Parkinson Disease"[MeSH Terms] OR Parkinson*[Title/Abstract]) | ("dystonia" OR "focal dystonia" OR "cervical dystonia" OR "generalized dystonia" OR blepharospasm OR "writer's cramp") (Title) and ("rating scale*" OR "clinical rating*" OR "clinical scale*" OR questionnaire* OR "assessment tool*" OR "outcome measure*" OR "Clinical Outcome Assessment" OR COA) (Title) and (validation OR psychometric* OR reliability OR validity OR clinimetric* OR responsiveness OR "sensitivity to change" OR review OR "systematic review") (Topic) not Parkinson (Topic) |
