# Supplementary material for: Clinical Outcome Assessments in Movement Disorders: A Scoping Review of Current Rating Scales and Future Needs Beyond Parkinson's Disease
Source: Mov Disord Clin Pract. 2026 Nov 6:10.1002/mdc3.70890. Online ahead of print. doi: 10.1002/mdc3.70890 (PMC13743828; doi:10.1002/mdc3.70890)
Supplement: Supplementary file 2 [file MDC3-9999-0-s002.docx]

**Supplementary Table 2**

| **Abbreviation** | **Definition** |
| --- | --- |
| **AGI** | Ataxia Global Initiative |
| **AIMS** | Abnormal Involuntary Movement Scale |
| **ARAT** | Action Research Arm Test |
| **ARQIP** | All India Institute of Medical Sciences RLS Questionnaire for Indian Patients |
| **ARSACS** | Autosomal Recessive Spastic Ataxia of Charlevoix-Saguenay |
| **A-TAC** | Autism-Tics, AD/HD and other Comorbidities inventory |
| **BAD** | Barry-Albright Dystonia scale |
| **BARS** | Brief Ataxia Rating Scale |
| **BBS** | Berg Balance Scale |
| **BDI-II** | Beck Depression Inventory-II |
| **CamQUAIT** | Cambridge Questionnaire for Apathy and Impulsivity Traits |
| **CBFS** | Cortical Basal Ganglia Functional Scale |
| **CCDRS** | Comprehensive Cervical Dystonia Rating Scale |
| **CDIP-58** | Cervical Dystonia Impact Profile |
| **CDQ-24** | Craniocervical Dystonia Questionnaire |
| **CGI-S** | Clinical Global Impression–Severity Scale |
| **CH-RLSq** | Cambridge–Hopkins Diagnostic Questionnaire for RLS |
| **CICS** | Cerebellar Impulsivity Compulsivity Scale |
| **CTTT** | Clinch Token Transfer Test |
| **DAT** | Digital Assessment Task |
| **DGI** | Dynamic Gait Index |
| **DIS** | Dyskinesia Impairment Scale |
| **DNMSQuest** | Dystonia Non-Motor Symptoms Questionnaire |
| **EBI** | Ethos Brief Index |
| **ECAS** | Edinburgh Cognitive and Behavioural ALS Screen |
| **FAB** | Frontal Assessment Battery |
| **FACR-HI** | Friedreich Ataxia Caregiver-Reported Health Index |
| **FALL-HD** | Fall Questionnaire for Huntington’s Disease |
| **FARS-ADL** | Friedreich’s Ataxia Rating Scale Activities of Daily Living |
| **FuRST 2.0** | Functional Rating Scale 2.0 |
| **FXTAS-RS** | Fragile X-Associated Tremor Ataxia Syndrome Rating Scale |
| **f-SARA** | Modified Functional Scale for the Assessment and Rating of Ataxia |
| **HADS** | Hospital Anxiety and Depression Scale |
| **HAT** | Hypertonia Assessment Tool |
| **HD-BQ** | Huntington’s Disease Behavioral Questionnaire |
| **HD-CAB** | Huntington’s Disease Cognitive Assessment Battery |
| **HD-CFRS** | Huntington’s Disease Cognitive Functional Rating Scale |
| **HDDMS** | HD Digital Motor Score |
| **HDQoL** | Huntington’s Disease Quality of Life |
| **HDQoL-C** | Huntington’s Disease Quality of Life for Caregivers |
| **HDQLIFE** | Huntington’s Disease Quality of Life End of Life Planning |
| **HD-HI** | Huntington’s Disease Health Index |
| **HD-PRO-TRIAD** | Huntington’s Disease Patient-Reported Outcomes Triad |
| **Hi-DEF** | Huntington’s Disease Everyday Functioning |
| **ICARS** | International Cooperative Ataxia Rating Scale |
| **INAS** | Inventory of Non-Ataxia Signs |
| **IPUTS** | Individualized Premonitory Urge for Tics Scale |
| **IRLS** | International Restless Legs Syndrome Study Group Rating Scale |
| **IRLSSG** | International Restless Legs Syndrome Study Group |
| **IS** | Irritability Scale |
| **KRLS-QoL** | Kohnen Restless Legs Syndrome Quality of Life |
| **LARS-s** | Short Lille Apathy Rating Scale |
| **LASSI-L** | Loewenstein-Acevedo Scale for Semantic Interference and Learning |
| **LIVECHART PRO** | LIVECHART Patient-Reported Outcome Tool |
| **MBS-ET** | Most Bothersome Symptom–Essential Tremor Questionnaire |
| **MD-CRS** | Movement Disorder–Childhood Rating Scale |
| **MDIB** | Movement Disorders Interpretation Bias Scale |
| **MDRS** | Mattis Dementia Rating Scale |
| **MDS** | Movement Disorders Society |
| **mFARS** | Modified Friedreich Ataxia Rating Scale |
| **MMSE** | Mini-Mental State Evaluation |
| **MoCA** | Montreal Cognitive Assessment |
| **MOVES** | Motor Tic, Obsessions and Compulsions, Vocal Tic Evaluation Survey |
| **MRVS** | Modified Rush Video-Based Tic Rating Scale |
| **m-RLS-DQ** | Modified RLS Diagnostic Questionnaire |
| **MSNQ** | Multiple Sclerosis Neuropsychological Screening Questionnaire |
| **PAS-ET** | Patient Attainment Scale–Essential Tremor |
| **PBA-s** | Problem Behaviors Assessment–Short Form |
| **PD-CRS** | Parkinson’s Disease Cognitive Rating Scale |
| **PIDS** | Pain in Dystonia Scale |
| **PMS** | Pearlin Mastery Scale |
| **PRAIS** | Person-Reported Ataxia Impact Scale |
| **PROM-Ataxia** | Patient-Reported Outcome Measure of Ataxia |
| **PROMIS** | Patient-Reported Outcomes Measurement Information System |
| **PSPRS** | Progressive Supranuclear Palsy Rating Scale |
| **PQoL Carers** | Parkinsonism Carer Quality of Life Questionnaire |
| **PTQ** | Parent Tic Questionnaire |
| **PUTS** | Premonitory Urge for Tics Scale |
| **Q-Motor** | Quantitative Motor Assessment |
| **QUEST** | Quality of Life in Essential Tremor Questionnaire |
| **RBANS** | Repeatable Battery for the Assessment of Neuropsychological Status |
| **RLS** | Restless Legs Syndrome |
| **RLS-H** | Hindi Restless Legs Syndrome Screening Tool |
| **RLS-QoL-Abetz** | Quality of Life Instrument for Restless Legs Syndrome (Abetz) |
| **RLSSQ** | Restless Legs Syndrome Screening Questionnaire |
| **RLSSCBQ** | Restless Legs Syndrome Self-Care Behaviour Questionnaire |
| **SARA** | Scale for the Assessment and Rating of Ataxia |
| **SARAhome** | SARA Home Version |
| **SARA-Le** | Scale for the Assessment and Rating of Ataxia Live e-Version |
| **SARA-TH** | Scale for the Assessment and Rating of Ataxia Thai Version |
| **SCACOMS** | Spinocerebellar Ataxia Composite Scale |
| **SEC** | Scientific Evaluation Committee |
| **SelfCog** | Digitized Cognitive Monitoring Battery |
| **SF-36** | 36-Item Short Form Health Survey |
| **SIBS-T** | Self-Injurious Behavior Scale for Tic Disorders |
| **SIS** | Snaith Irritability Scale |
| **SODA** | Scale for Ocular Motor Disorders in Ataxia |
| **SPAS** | Sensory Phenomenon Assessment Scale |
| **SPAXCOM** | Spastic Ataxia Composite |
| **SPRS** | Spastic Paraplegia Rating Scale |
| **SQST** | Single-Question Screening Tool |
| **SRSQ** | Social Relationship Self-Questionnaire |
| **STEPS** | Step Test Evaluation of Performance on Stairs |
| **STSSS** | Shapiro Tourette Syndrome Severity Scale |
| **TAPS** | Technical Ability and Performing Scale |
| **TDS** | Tourette Disorder Scale |
| **TDSC** | Tourette Discrimination-Stigmatization Scale |
| **TETRAS** | Essential Tremor Rating Assessment Scale |
| **TETRAS-PRO** | Essential Tremor Rating Assessment Scale Patient-Reported Outcome |
| **TMT** | Tinetti Mobility Test |
| **TSCGI** | Tourette Syndrome Clinical Global Impression |
| **TWSTRS** | Toronto Western Spasmodic Torticollis Rating Scale |
| **UHDRS** | Unified Huntington’s Disease Rating Scale |
| **UHDRS-TMS** | Unified Huntington’s Disease Rating Scale Total Motor Score |
| **ULTTT** | Upper Limb Token Transfer Test |
| **UPDRS** | Unified Parkinson’s Disease Rating Scale |
| **USDRS** | Unified Spasmodic Dysphonia Rating Scale |
| **WHODAS 2.0** | World Health Organization Disability Assessment Schedule 2.0 |
| **YGTSS** | Yale Global Tic Severity Scale |
