# Supplementary material for: Clinical Outcome Assessments in Movement Disorders: A Scoping Review of Current Rating Scales and Future Needs Beyond Parkinson's Disease
Source: Mov Disord Clin Pract. 2026 Nov 6:10.1002/mdc3.70890. Online ahead of print. doi: 10.1002/mdc3.70890 (PMC13743828; doi:10.1002/mdc3.70890)
Supplement: Supplementary file 3 [file MDC3-9999-0-s003.docx]

**Suppl Fig. 1** **Flowchart diagram for studies included in the review**

**Identification of studies via databases**

Records removed *before screening*:

Duplicate records removed (n = 443)

Records identified from:

Pubmed (n = 967)

Web of Science (n = 1253)

**Identification**

Records excluded

(n = 1161 )

Records screened

(n = 1777)

**Screening**

Reports assessed for eligibility

(n = 616)

Studies included in review

(n = 261)

**Included**

Source: Page MJ, et al. BMJ 2021;372:n71. doi: 10.1136/bmj.n71.

This work is licensed under CC BY 4.0. To view a copy of this license, visit <https://creativecommons.org/licenses/by/4.0/>
